# High-energy resolution X-ray spectroscopy at actinide M_4,5_ and ligand K edges: what we know, what we want to know, and what we can know

**DOI:** 10.1039/d1cc04851a

**Published:** 2021-11-16

**Authors:** Kristina O. Kvashnina, Sergei M. Butorin

**Affiliations:** The Rossendorf Beamline at ESRF, The European Synchrotron CS40220 38043 Grenoble Cedex 9 France kristina.kvashnina@esrf.fr; Institute of Resource Ecology, Helmholtz Zentrum Dresden-Rossendorf (HZDR) PO Box 510119 01314 Dresden Germany; Department of Chemistry, Lomonosov Moscow State University Moscow 119991 Russia; Condensed Matter Physics of Energy Materials, X-ray Photon Science, Department of Physics and Astronomy, Uppsala University P.O. Box 516 SE-751 20 Uppsala Sweden sergei.butorin@physics.uu.se

## Abstract

In recent years, scientists have progressively recognized the role of electronic structures in the characterization of chemical properties for actinide containing materials. High-energy resolution X-ray spectroscopy at the actinide M_4,5_ edges emerged as a promising direction because this method can probe actinide properties at the atomic level through the possibility of reducing the experimental spectral width below the natural core-hole lifetime broadening. Parallel to the technical developments of the X-ray method and experimental discoveries, theoretical models, describing the observed electronic structure phenomena, have also advanced. In this feature article, we describe the latest progress in the field of high-energy resolution X-ray spectroscopy at the actinide M_4,5_ and ligand K edges and we show that the methods are able to (a) provide fingerprint information on the actinide oxidation state and ground state characters (b) probe 5f occupancy, non-stoichiometry, defects, and ligand/metal ratio and (c) investigate the local symmetry and effects of the crystal field. We discuss the chemical aspects of the electronic structure in terms familiar to chemists and materials scientists and conclude with a brief description of new opportunities and approaches to improve the experimental methodology and theoretical analysis for f-electron systems.

## Abstract 2

More than 10 years have passed since the first uranium M4 HERFD and RIXS experiments in 2009. In this feature article, we describe the progress in actinide M4,5 HERFD and RIXS techniques for probing the actinide electronic structure.

## Graphical abstract

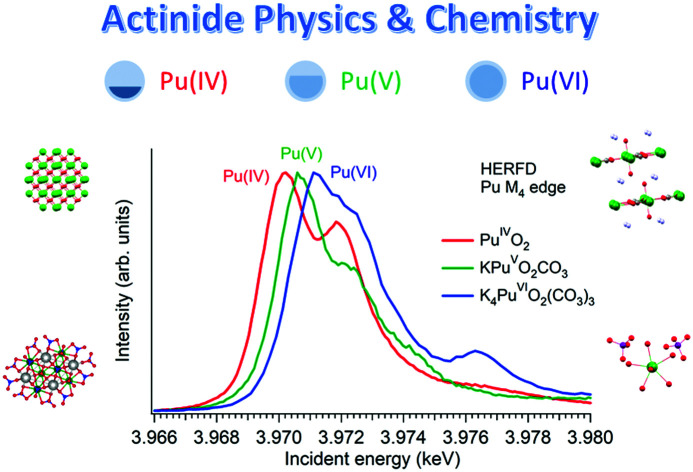

## Introduction

Most of the radioactive chemical elements are situated at the bottom of the periodic table and form the actinide group. These elements are considered to be ranging from atomic number 89 to 103 and play a key role in nuclear chemistry with important applications in the fields of nuclear energy and environmental science. As the atomic number increases in the actinide series, the added electrons enter the 5f shell in the ground state configuration. Most of the fascinating properties of actinide materials are related to the partially filled 5f valence shell, and in contrast to compounds of other elements from the periodic table, they are poorly understood. This includes their surprising reactivity, magnetic and crystal structure properties and rather unpredictable covalent or ionic nature of their bonds.

Probing the behaviour of electrons requires experimental techniques that are non-sensitive to contamination at the surface of the sample being studied. The experimental work presented here strongly benefited from the existence of the large-scale facilities and the synchrotron radiation beamlines with the high instrumental resolution achieved in the last few years. Synchrotron radiation sources offer a number of unique advantages for X-ray spectroscopy experiments. One of them is the element-selectivity using the energy of incident X-rays, which can be tuned and thus allows one to probe the core and valence transitions. Due to the selection rules, the electric dipole transitions from the ground state reach only a particular number of final states, providing a fingerprint of the ground state configuration. At the same time the effects of the charge transfer excitation, crystal field splitting, electron–electron interactions and hybridization between molecular orbitals can be studied in detail. Recent developments of high energy resolution setups for X-ray spectroscopic studies including X-ray emission spectrometers^1,2^ have given the unique opportunity to detect and resolve those effects.

More than 10 years have passed and more than 100 papers have been published after the first X-ray spectroscopy experiment in the high-energy resolution mode on uranium systems at the U M_4_ edge (∼3728 eV)^3^ at the ID26 beamline of ESRF in 2009,^4,5^ where an X-ray emission spectrometer was utilized. Quite a bit is known for the moment and X-ray absorption spectroscopy (XAS) and X-ray absorption near edge structure (XANES) in the high-energy resolution fluorescence detection (HERFD) mode (also known as HR-XANES) together with resonant inelastic X-ray scattering (RIXS) or resonant X-ray emission spectroscopy (RXES) are now common techniques for probing the actinide electronic structure and for studying the physics and chemistry of the f-block elements.^6^ More beamlines at various synchrotron facilities were employed to construct the experiment stations for the tender X-ray energy range and to equip them with X-ray emission spectrometers.^7,8^ All these efforts will surely be beneficial in terms of understanding the mechanisms of chemical reactions involving actinides at the atomic level, for which RIXS and HERFD methods are employed in the tender M_4,5_ X-ray energy range (3500–4000 eV). It should be noted that actinide 5f states can be also probed with X-ray spectroscopy at the N_4,5_ edges^9–11^ (4d–5f transitions in the ∼700–800 eV range) and at the O_4,5_ edges (5d–5f transitions in the ∼90–150 eV range).^10,12–16^ However, such measurements require the use of a soft-X-ray beamline and vacuum environment around the sample, which might be challenging to access highly radioactive materials. X-ray spectroscopy at the actinide L_3_ edge benefits from the high penetration depth in the hard X-ray energy range (∼17 000–18 000 eV) but it probes the actinide 6d states (2p–6d transitions),^6^ so do measurements at N_6,7_ edges^9^ (4f–6d transitions in the ∼300–500 eV range). However, the large core-hole lifetime broadening at the L_3_ edge smears out the spectral structures. Furthermore, the 6d states are not as well enough hybridized with the 5f states as, for example, ligand 2p states to reflect the changes in the 5f state profile.

In this article we show how the XANES, HERFD and RIXS methods at the actinide M_4,5_ edges and ligand K edge can provide an unprecedented amount of detail regarding the actinide oxidation state, speciation, defects, nature of the actinide chemical bonding, 5f occupation and degree of the 5f localization. Such fundamental knowledge is a key step towards solving the extreme complexity of the chemistry problems with radionuclides,^17–26^ safe disposal of nuclear wastes and prediction of the radionuclide behaviour in the environment.^17,27–31^

## What we know

A brief introduction to HERFD-XANES, RIXS and XES is given in the next section. More information about these techniques can be found in the cited literature.^32,33^

### HERFD-XANES, RIXS and XES methods

**Fig. 1:**
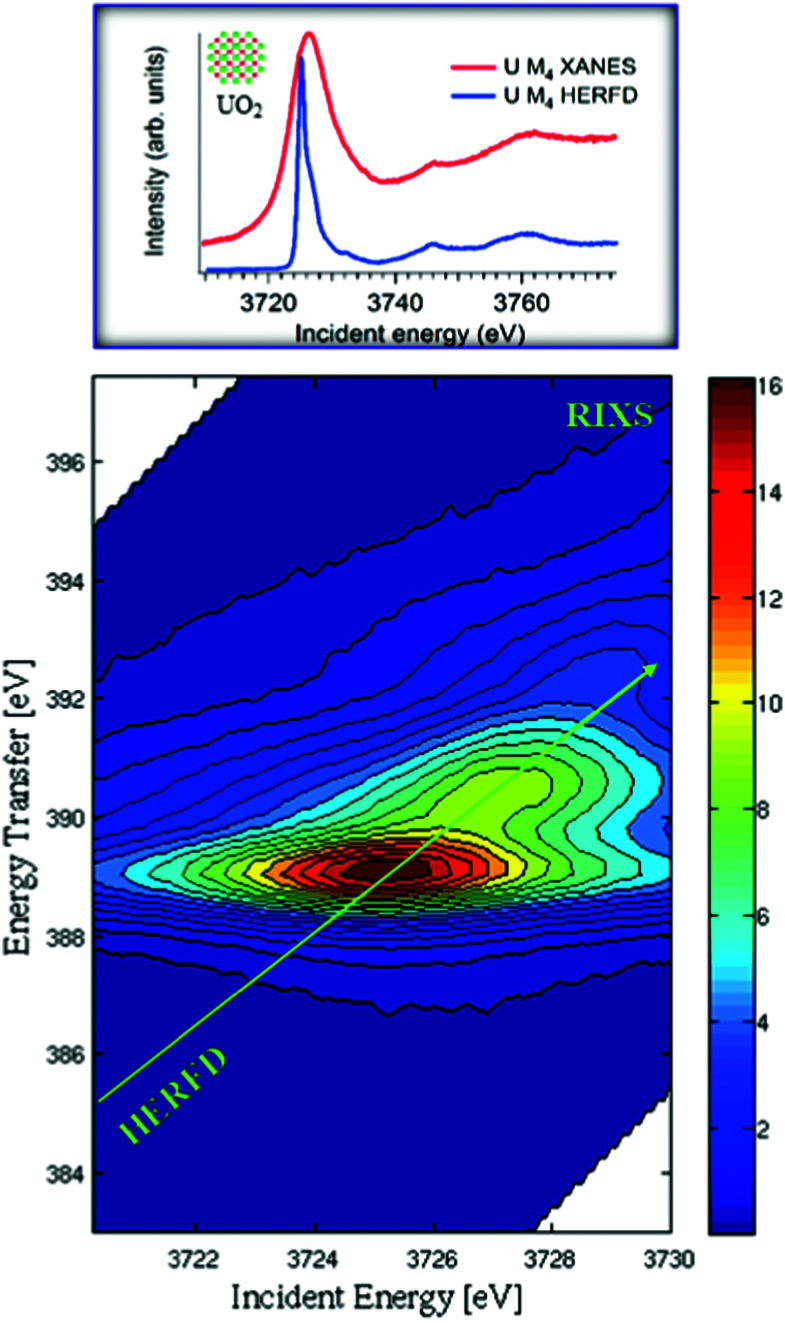
XANES, HERFD and core-to-core 4f–3d RIXS measurements at the U M_4_ edge for UO_2_. RIXS data are shown as a contour map in a plane of incident and transferred photon energies, where the vertical axis represents the energy difference between the incident and the emitted energies. Variations of the color in the plot are related to different scattering intensities. The HERFD spectrum corresponds to a diagonal cut through the RIXS plane at the maximum of the Mβ emission line.

In conventional XANES experiments, the large core-hole lifetime broadening, which is ∼4 eV^34,35^ for the actinide M_4_ edges, gives rise to broad spectra.^36^ A spectral narrowing below the natural core hole lifetime width can be achieved by employing an X-ray emission spectrometer and recording the HERFD spectra. Fig. 1 illustrates the mechanism of the sharpening effect in the HERFD measurements at the U M_4_ edge.

The main edge of the U M_4_ XANES spectrum arises from the electronic transitions from the U 3d_3/2_ to 5f level. The U M_4_ edge absorption features can be recorded in the HERFD mode with the help of a Si(220) crystal analyzer, installed in the X-ray emission setup. The emission spectrometer is tuned to the Mβ (4f_5/2_–3d_3/2_) XES transition and the XANES spectrum is recorded by monitoring the emission energy signal at the maximum of the Mβ intensity as a function of incident energy. The advantage of such a setup is that the width of the spectral features is no longer defined by the 3d_3/2_ core-hole lifetime but by the smaller 4f_5/2_ core-hole lifetime broadening in the final state of the spectroscopic process. From the experimental point of view, the XES spectra are recorded by scanning the emitted energy with the crystal analyzer, while keeping the incident energy fixed. If the incident energy is selected above the XANES region, non-resonant XES spectra are recorded. If the incident energy is selected below or near the XANES region, RIXS or RXES spectra are recorded.

Fig. 1 shows the RIXS data as a contour map in a plane of incident and transferred photon energies. The horizontal axis is identical to the energy range of the XANES spectrum. The vertical axis represents the energy difference between the incident and the emitted energies. The emitted energies have been selected across the Mβ XES line. The typical energy range along the vertical axis of RIXS is ±10 eV near the main Mβ XES maximum. Variations of colour in the plot is related to different scattering intensities. The HERFD spectrum corresponds to a diagonal cut through the RIXS plane at the maximum of the Mβ emission line.

### X-ray emission spectrometer in Johann configuration

All the high-energy resolution techniques mentioned above become available using the X-ray emission spectrometer setup. Technically, an X-ray emission spectrometer consists of a crystal analyzer and a detector, which can be realized with different geometries. The most known types are the Johansson,^37^ Johann^38^ and von Hamos^39^ geometries. The existence of various geometries provides an opportunity to use the most appropriate instrument for a particular experimental station. In cases of the Johann and Johannson geometries, a point-to-point focusing scheme is realized, where both the crystal analyzer and the detector are moved when the emitted energy is scanned.^40–47^ This scheme provides a higher detection signal, better energy resolution and covers a larger solid angle. In contrast, the possibility of recording the emission lines with the energy dispersive (von-Hamos) geometry^48^ and dispersive Rowland cycle geometry^49,50^ is very practical since it does not include movable parts.

**Fig. 2:**
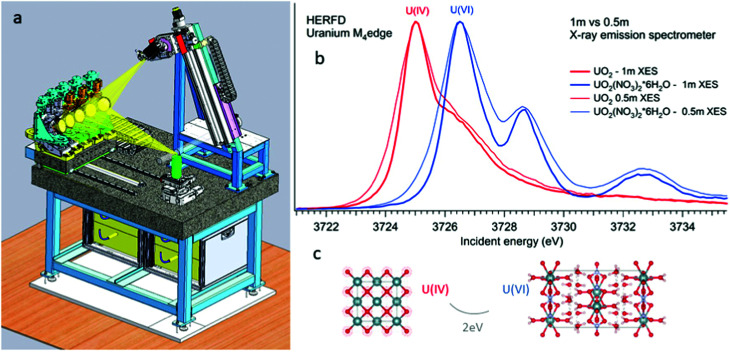
Schematic drawing of the five-crystal X-ray emission spectrometer at the BM20 of ESRF (left). Experimental U M_4_ HERFD of UO_2_ and UO_2_(NO_3_)_2_·*6H_2_O recorded with 0.5 m and 1 m bending radius of crystal-analyzers of the X-ray emission spectrometer.

Fig. 2 shows a schematic drawing of the Johann-type point-focusing X-ray emission spectrometer^1,2^ with five spherically bent crystal analyzers installed at the BM20 beamline^51^ of ESRF. The spectrometer at the BM20 beamline can be operated with crystal analyzers with 0.5 m and 1 m bending radii.^52^ Compared to standard 1 m setups and analyzers, an intensity gain (factor of 10) is obtained with 0.5 m setup at the U M_4_ edge. The decrease in the resolution is noticeable since the Bragg angle of the analyser is at 75° (Fig. 2 right). The detected energy shifts between tetravalent and hexavalent uranium in the HERFD data will be discussed in detail in the next section. The 0.5 m setup can be used in situations when the acquisition time has to be minimized and in studies of diluted actinide species, probing the actinide concentrations down to a ppm level. The 1 m setup gives better energy resolution and should be used in cases when tiny absorption features have to be detected with a high precision.

**Fig. 3:**
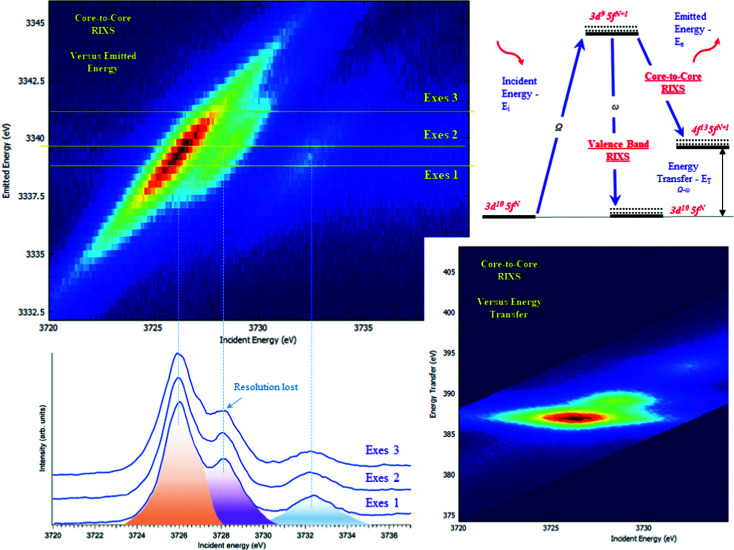
The core-to-core 3d–4f RIXS of UO_2_(NO_3_)_2_·*6H_2_O recorded near the maximum of the U Mβ emission line and plotted in two ways: (top-left) in a plane of incident (*E*_i_) and emitted energies (*E*_e_) or (bottom-right) in a plane of incident (*E*_i_) and transferred photon energies (*E*_T_), where the vertical axis represents the energy difference between the incident (*E*_i_) and the emitted energies (*E*_e_). The corresponding cuts through the RIXS plane at different emission energies (HERFD scans) (bottom-left) and the energy diagram of the core-to-core and valence-to-core processes (top-right).

The typical procedure to perform high-energy resolution studies is to record the RIXS map around the selected emission energy and analyze the RIXS map.^3,6,53–58^Fig. 3 shows the RIXS map recorded for the UO_2_(NO_3_)_2_·*6H_2_O at the BM20^51^ beamline of ESRF. RIXS data can be shown as contour maps in two formats: (a) as a plane with incident (*E*_i_) and emitted energy (*E*_e_) axes (Fig. 3 top) or (b) as a plane with incident (*E*_i_) and transferred photon energy (*E*_T_) axes, where the vertical axis represents the energy difference between the incident (*E*_i_) and the emitted energies (*E*_e_). During the analysis, special attention has to be paid to the features that do not lie on the diagonal cut^54^ (related to HERFD). If all the features are situated along the diagonal direction, the HERFD measurements can be performed by recording the one-dimensional scan.

The energy resolution of the HERFD data depends on the selected emission energy. Fig. 3 (bottom) shows the HERFD data recorded at the maximum of the non-resonant XES (marked as Exes 1), at the maximum of the resonant XES (marked as Exes 2) and at an emission energy above Exes 2 (marked as Exes 3). Visual inspection of the recorded features shows the loss in the energy resolution for the HERFD spectra recorded at Exes 2 and Exes 3. Our experience shows that the best energy resolution in the HERFD data is obtained when it is recorded at the maximum of the non-resonant X-ray emission line. Non-resonant Mα and Mβ XES spectra have the low intensity as compared to the resonant excited one but in order to obtain the best possible resolution it is worth spending time and effort to record XES data with high statistics and to select a XES maximum for the HERFD mode.

RIXS measurements at the core lines (Mα, Mβ, *etc.*) are usually referred to as core-to-core RIXS.^6,57^ On the other hand, a direct involvement of the valence 5f electrons in the spectroscopic process is probed by valence-to-core or valence band RIXS.^14,53,57,59^ The intermediate state for core-to-core and valence-to-core RIXS measured with excitation at actinide 3d edges is the same and includes a created 3d core-hole, making the technique element, site and symmetry selective. The energy transfer in the core-to-core RIXS process at the Mβ emission line is relatively large (∼390 eV) and there is a 4f core-hole in the final state. In the core-to-valence RIXS process, the energy transfer is only a few eV, and no core-hole is present in the final state with a valence 5f electron excited into the unoccupied state.^6,57^

## What we want to know

The next four sections describe the information which can be obtained from the actinide M_4,5_ edge HERFD-XANES and RIXS data.

### Oxidation state verification

**Fig. 4:**
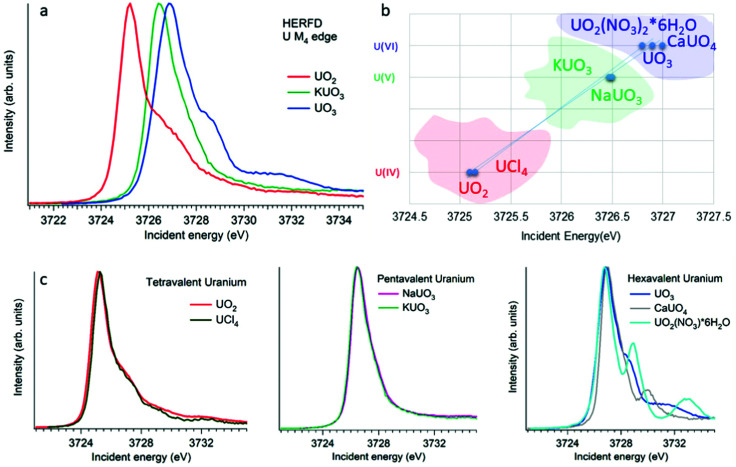
(a) Uranium M_4_ HERFD data of UO_2_, KUO_3_ and UO_3_ with U(iv), U(v) and U(vi) oxidation states, respectively.^3,6,76^ (b) The position of the HERFD peak for several U-containing systems in the order of the increasing energy and oxidation state. (c) U M_4_ HERFD data^3,6,56^ for several tetravalent, pentavalent and hexavalent systems in different crystal structures.

XANES spectroscopy characterizes the chemical state of the absorbing atom. The actinide M_4,5_ HERFD-XANES method gives the opportunity to fingerprint the oxidation state of the actinide-containing materials. The first U M_4_ experiment has been performed on the mixed uranium oxides,^3^ which was followed by several other studies on the chemical state verification in actinide systems.^3,6,9,12,60–77^Fig. 4a shows the HERFD data at the U M_4_ edge of the UO_2_, UO_3_ and KUO_3_, published by Leinders and co-workers^76^ as a representative example of the oxidation state detection in tetravalent (UO_2_), hexavalent (UO_3_) and pentavalent (KUO_3_) uranium systems. Similar trends were observed later for the plutonium^61–64^ and neptunium^71^ systems in different chemical states.

The HERFD spectrum of UO_2_ exhibits the absorption maximum at ∼3725 eV with a shoulder around 3727 eV, which has been assigned to the contribution of the multiplet structure of the U 5f states.^3,54^ The position of the HERFD peak for the pentavalent KUO_3_ and hexavalent UO_2_(NO_3_)_2_·*6H_2_O systems was found to be at 3726.5 and 3727 eV, respectively. The energy position of the HERFD maximum corresponds to the energy difference between the 3d and 5f levels. As soon as the 5f shell gains or loses more electrons the chemical shift in XANES can be detected. It should be noted that the chemical shift between various oxidation states in the row U(iv)–U(v)–U(vi) does not show a linear change. The difference in the HERFD peak position between U(iv) and U(v) or U(vi) is much greater than the one between U(v) and U(vi) (Fig. 4b).

Moreover, the energy position of the U M_4_ HERFD spectra can be strongly influenced not only by the chemical U state but also by the coordination geometries and ligand field splitting of the 5f shell. However, the detectable difference will be in the order of 0.1 eV *versus* 1–2 eV which is observed for various U oxidation states. Fig. 4b summarizes the position of the HERFD peak for several U-containing systems in the order of increased energy and oxidation state. It shows that chemical state verification still can be done even in the presence of various geometries and ligands around the absorbing U atom.

Fig. 4c reports the U M_4_ HERFD data for tetravalent, pentavalent and hexavalent species. In the case of tetravalent species, the energy position of the peak maximum increases on going from UO_2_ to UCl_4_ and is related to the ability of an element to attract the bonded electrons towards itself or in other words related to the electronegativity variation. In the periodic table the electronegativity increases from the left to the right side, which also depends on the atomic size. The closer connection of the electrons to the nucleus of an atom (for smaller ion) leads to more interactions between them. Thus, an increase in the atomic size leads to less attraction of electrons from the ligand to the uranium atom. The difference in the energy position of the HERFD peak between KUO_3_^76^ and NaUO_3_^56^ is almost negligible, but noticeable. The best example to see the influence of the crystal structure on HERFD in the presence of the same U(vi) oxidation state is shown in Fig. 4c (right). We can see in this figure that not only the energy position of the HERFD peak but also the shape changes significantly.^78^ We discuss the effects of the crystal structure influence in the next section.

The identification of the oxidation state by HERFD has become important for many areas of actinide science since it provides information on how electrons are distributed between the elements. The oxidation–reduction (redox) reactions involve the transfer of electrons between chemical species.^3,60,63,66,68,69,72,76,77,79^ Many biological processes rely on the electron transfer. The environmental behaviour of actinides is characterized by a broad complexity in chemical species occurring in different environments and varies as a function of the chemical and geochemical boundary conditions. The oxidation state of actinides controls the solubility of solid actinide species. For example, Pu(iv) species are considered less mobile than species with other oxidation states because of their solubility. Therefore, the HERFD method at the An M_4,5_ edges is the only one for the moment, which allows us to fingerprint the mixtures of actinide oxidation states with high precision (as low as 2% contribution) in environmentally relevant systems.^65,67,75,80,81^

Oxidation state is significant in the nanoscience field, which is rapidly growing and developing in various scientific communities. Structures with nanoscale dimensions offer novel properties and lead to technologically new applications. One of the crucial aspects here is that the physical and chemical properties of the materials are changing as it comes to the nanoscale. The HERFD methodology at the actinide M_4,5_ edges has been successfully used in recent electronic structure investigations of the UO_2_,^66^ ThO_2_^82^ and PuO_2_^61,63^ nanoparticles (NPs).

**Fig. 5:**
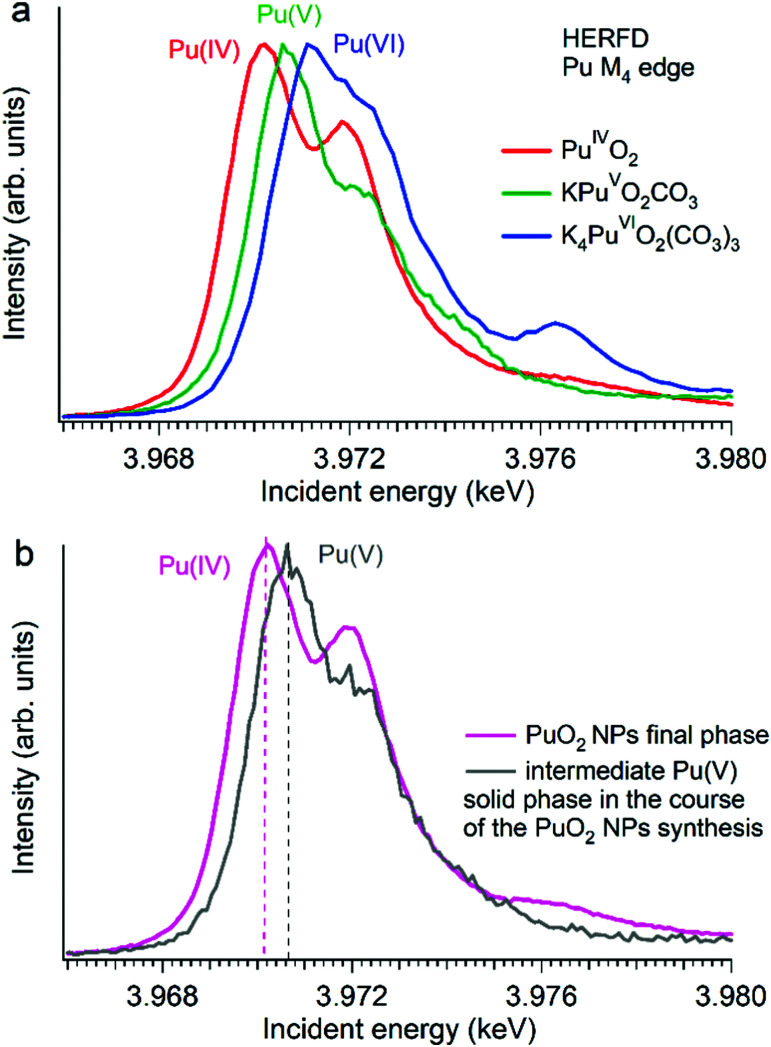
Plutonium M_4_ HERFD data (a) of PuO_2_, KPuO_2_CO_3_ and K_4_PuO_2_(CO_3_)_3_ with Pu(iv), Pu(v) and Pu(vi) oxidation states, respectively,^61,64^ and (b) of two plutonium phases obtained during the synthesis of PuO_2_ NPs from a Pu(vi) precursor at pH 11.^61^

Several reports indicated the high importance of the fundamental understanding of the PuO_2_ NPs in the context of environmental behaviour. The most intriguing question concerning the PuO_2_ NPs is the potential presence of different Pu oxidation states (*cf.*Fig. 5). A formation of the PuO_2_ NPs under various synthesis conditions (pH value and different initial solutions) has been recently investigated by Pu M_4_ HERFD. It has been shown that all spectral features of the PuO_2_ NPs are very similar to those of the PuO_2_ reference and were well reproduced by calculations based on the bulk PuO_2_ crystal structure.^63^ These NP studies lead to the discovery of a new plutonium compound (refined NH_4_PuO_2_CO_3_) with an unexpected pentavalent oxidation state.^61^

Authors experimentally traced the route of the Pu(vi) to PuO_2_ NP transformation as a two-step process.^61^ During the first few minutes the formation of the intermediate pentavalent phase was observed. Later, after several weeks of the precipitation reaction, the final product – PuO_2_ in 2 nm size – was formed. The Pu M_4_ HERFD data of both products reveal that intermediate and final phases have Pu in different oxidation states according to the position of their white line^61^ (Fig. 5 bottom). This has led to the new knowledge concerning the formation of the Pu-containing NPs in aqueous solutions, which is extremely important for the performance assessment of geological repositories of the spent fuel and radioactive waste as well as for the effective remediation of contaminated sites.

Recently, highly crystalline UO_2_ NPs with a size of 2–3 nm were studied by means of U M_4_ HERFD.^66^ The results also confirm that U(iv) is the dominant oxidation state. The similarity of the uranium and plutonium oxide NPs is important for environmental chemistry as both are mobile in their colloid form. However, there is still a significant difference in the stability of the UO_2_ NPs *versus* PuO_2_ NPs. It was shown that the PuO_2_ NPs remain intact after several months^63^ with respect to the size (∼2 nm) and oxidation state.^63^ Contrary to that, the UO_2_ NPs increase in size up to ∼6 nm and become partially oxidized with time even if the samples were kept under inert conditions.^66^ It was found that the U(v) signal increased and a conversion to the U_4_O_9_ phase took place. Moreover, the effect of the X-ray beam exposure has been detected. Overall, it was shown that the UO_2_ NPs are more reactive compared to the PuO_2_ NPs.^66^ Therefore, actinide M_4,5_ HERFD has an impressive capacity to detect the presence of tiny impurities of different oxidation states, which is imperative for the actinide nanoscience field.

### 5f occupancy, covalency and charge-transfer effects

Besides the formal oxidation state of actinides, the actual population of the 5f shell is an important quantity which affects the physical and chemical properties of actinide compounds.

The actual 5f occupancy is rather non-integer in contrast to the case of the description/notation of the formal oxidation state and depends on the covalency degree of the chemical bonding and on the strength of the charge-transfer effects between actinide and ligand atoms as a result of hybridization of the actinide 5f states with ligand states. These effects lead to appearance of so-called charge-transfer satellites in the X-ray absorption spectra at the 3d edge of actinides. The improved resolution due to a reduced core-hole lifetime broadening of the HERFD spectra allows for resolving such satellites.

**Fig. 6:**
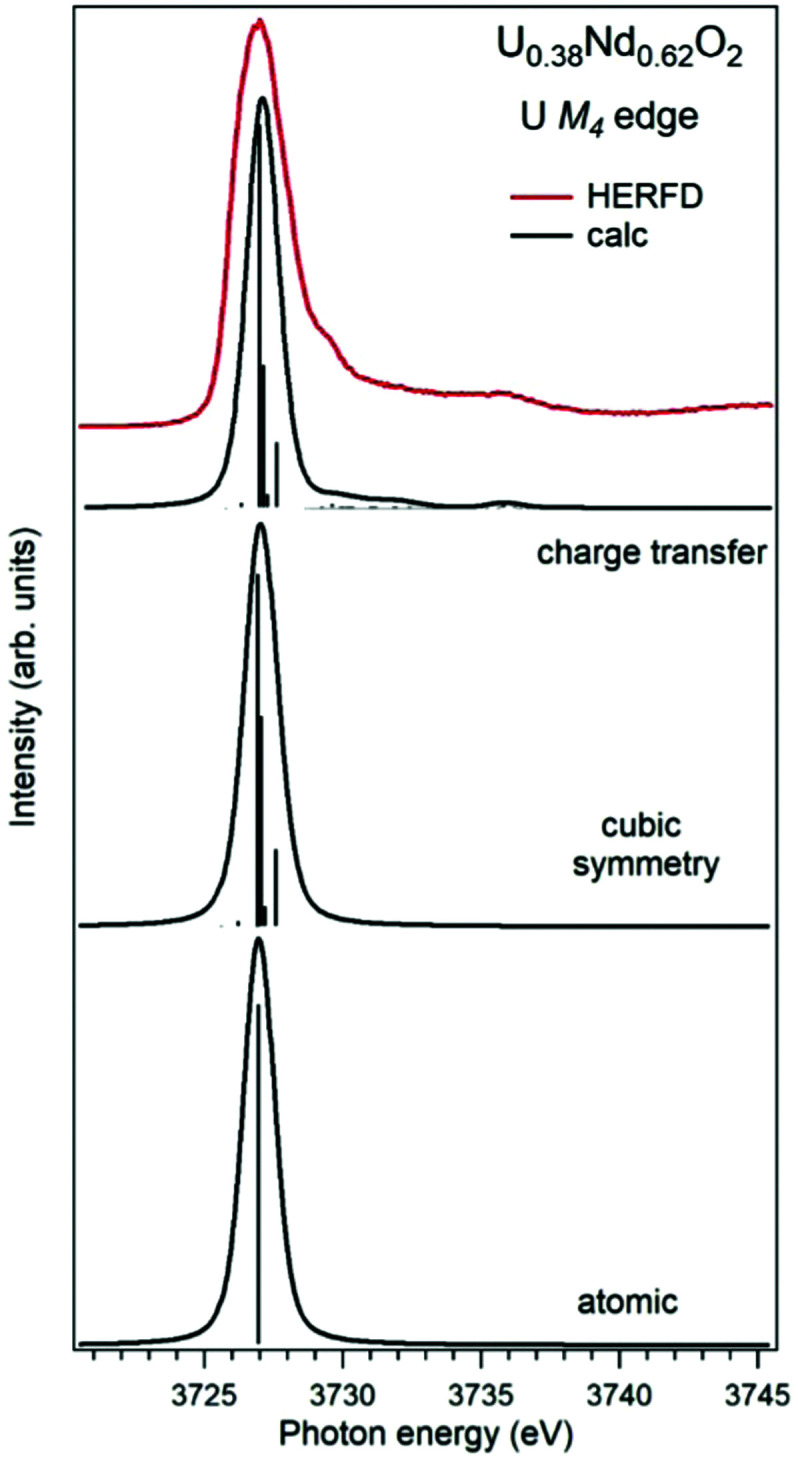
Experimental^77^ and calculated XAS spectra at the U M_4_ edge of U_0.38_Nd_0.62_O_2_. The spectra are calculated using atomic and crystal-field multiplet theory for the U^6+^ ion and Anderson impurity model, respectively.

Establishing the energy positions of the charge-transfer satellites and their relative intensity with respect to the main line helps in estimating the hybridization strength *V* of the actinide 5f states with ligand states, the value of the charge-transfer energy *Δ* and, consequently, the 5f occupancy n_f_ using the Anderson impurity model (AIM).^83^ Such an approach was successfully applied for the HERFD studies of UO_2_,^9^ U_1−*x*_La_*x*_O_2_^74^ and uranates with U(v) and U(vi).^56^ As an example, Fig. 6 shows a comparison of the U M_4_ HERFD spectrum of U_0.38_Nd_0.62_O_2_^77^ where uranium was found to be mainly in the U(vi) chemical state and in the cubic environment. The recorded spectrum is compared with the results of calculations using atomic multiplet and cubic crystal-field multiplet theory and AIM, respectively.

Within the atomic- and crystal-field multiplet theory, the spectra were calculated for transitions between 5f^0^ and 3d^9^5f^1^ configurations. The construction of the ground and final state Hamiltonians was carried out as described in ref. 9 as well as the equation used to calculate the XAS spectra. The required Slater integrals, spin–orbit coupling constants and matrix elements were obtained with the TT-MULTIPLETS package which combines Cowan's atomic multiplet program^84^ (based on the Hartree–Fock method with relativistic corrections) and Butler's point-group program,^85^ which were modified by Thole.^86^ The Slater integrals were reduced to 80% of their *ab initio* Hartree–Fock values in the calculations of the spectra and the Wybourne's crystal-field parameters for the 5f shell in the cubic (*O*_h_) symmetry were set to *B*^4^_0_ = −0.93 eV and *B*^6^_0_ = 0.35 eV, which are similar to those for pure UO_2_.^9^

The AIM calculations were performed in a similar manner as described in ref. 59 and 87. The model parameter values were chosen to be the same as in ref. 56 for Pb_3_UO_6_ and their values were as follows: energy for the electron transfer from the O 2p band to the unoccupied U 5f states *Δ* = 0.8 eV; 5f–5f Coulomb interaction *U*_ff_ = 3.0 eV; 3d core-hole potential acting on the 5f electron *U*_fc_ = 5.0 eV and U 5f–O 2p hybridization term *V* = 1.2 eV (0.9 eV) in the ground (final) state of the spectroscopic process. A linear combination of the 5f^0^ and 5f^1^ν^1^ configurations was used to describe the ground state of the spectroscopic process and the final state was represented by a combination of 3d^9^5f^1^ and 3d^9^5f^2^ν^1^, where ν stands for an electronic hole in the O 2p band.

In contrast to the atomic and crystal-field multiplet approach, the AIM calculations reproduce the charge-transfer satellite in the U M_4_ HERFD-XAS spectrum of U_0.38_Nd_0.62_O_2_, which appears at around 3736 eV (about 9 eV above the main line). The calculations show that the contribution of the 3d^9^5f^2^ν^1^, configuration in the ground state is 41% which results in an *n*_f_ value equal to 0.41 electron. This value is smaller than, for example, *n*_f_ in another U(iv)-containing compound Pb_3_UO_6_ which was AIM-estimated using the octahedral symmetry and contribution of three electronic configurations in the ground state. The difference in n_f_ values means the difference in the covalency degree of the U 5f–O 2p chemical bonding which was found to be lower for U in the cubic symmetry in U_0.38_Nd_0.62_O_2_.

Besides oxides as representatives of the actinide solid-state systems with an extensive band structure, the oxidation state verification and covalency effects are important and are equally addressed in the field of molecular actinide coordination complexes.^18–21,68,88,89^ The actinide–ligand bonding is currently of high interest which is reflected in a number of theoretical and experimental studies, where scientists study the nature of An bonding in novel molecular systems, such as uranocene, thorocene, An-containing sandwich complexes, *etc.*, by X-ray spectroscopic methods at the ligand K-edges (*cf.* ref. 90 and references therein) and at the actinide M_4,5_ edges.^68,88^ These spectroscopic techniques make important contribution to the fast development of molecular non-aqueous actinide chemistry.

### Local symmetry influence

**Fig. 7:**
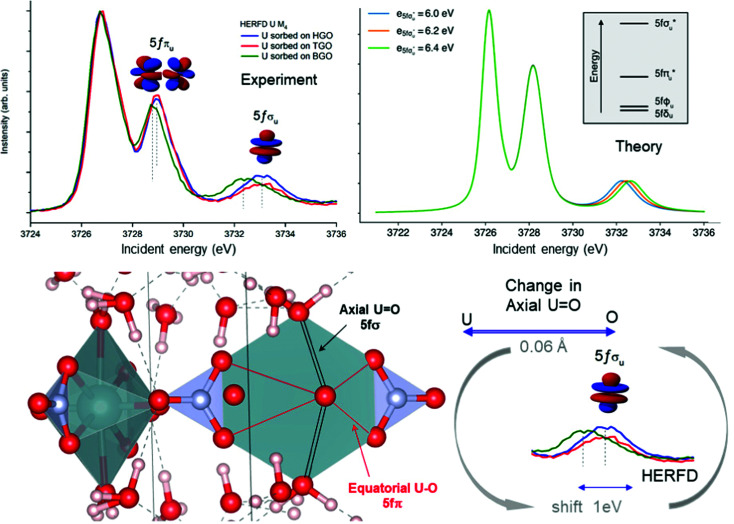
(left top) U M_4_ HERFD-XANES recorded for U(vi) sorbed onto GO synthesized by Hummers (HGO), Tour (TGO) and Brodie (BGO) methods. (right top) Theoretical U M_4_ HERFD spectra calculated using the Crispy code. (bottom) Uranyl structure and schematic representation of the axial and equatorial U–O bonds and changes seen by the HERFD method.

Local symmetry and changes of the crystal parameters influence the HERFD spectral shape.^78^ One typical example of such a crystal structure influence can be studied from the case of hexavalent uranium species. Fig. 2 (right top) shows the HERFD data of the hexavalent UO_2_(NO_3_)_2_·*6H_2_O. The spectrum contains three primary peaks separated by 2 and 4 eV between each other on the incident energy scale. These peaks reflect the splitting of the U 5f unoccupied states and were assigned to transitions to the nonbonding 5fδ_u_ and 5fφ_u_, antibonding 5fπ_u_*, and antibonding 5fσ_u_* molecular orbitals.^8,91–94^ The corresponding transitions are shown in Fig. 7, where more accessible information can be found. As seen before, the second peak provides information about equatorial U–O bonds and the third one provides information on U–O axial bonds.^92^ Previous M_4_ HERFD measurements of uranyl in lithium borate glasses show that a 0.06 Å change of the bond length can result in a shift of the third peak (fσ) by as much as 1 eV.^8^

We show below that HERFD at the actinide M_4_ edge is a powerful tool in the investigation of chemical reactions, where the interaction between actinide and other materials takes place. Recently, HERFD has been used to study the sorption mechanisms of radionuclides on graphene oxide (GO)^93^ and defect-rich graphene oxide surface.^92^Fig. 7 shows the experimental U M_4_ HERFD data^92^ recorded on GOs synthesized by Hummers's (HGO), Brodie's (BGO) and Tour's (TGO) methods. It was observed that BGO has a lower sorption capacity and lower activity compared to HGO and TGO in the case of U(vi).^93^ The lower sorption of the radionuclides by BGO correlates with the smaller relative amounts of carboxyl groups and smaller overall oxidation degree of this material.

The HERFD data reported in Fig. 7 (left panel) show a small shift of the absorption features of U(vi) sorbed onto GO synthesized using three different methods: HGO, TGO, and BGO, thus confirming the high sensitivity of this method to the elongation of the bond length. A small 0.01 Å elongation of the uranyl bond was found for the BGO material using EXAFS^93^ and indeed, the third peak in the M_4_ edge spectrum shows a shift of 0.7 eV to the lower energy.

An increase of the U–O axial bond distance results in the energetic stabilization of the 5fσ_u_* orbital, which, according to the calculations, shifts the third peak to lower energies, in agreement with the experimental observations. Fig. 7 (right panel) shows the uranium M_4_ HERFD spectra calculated using Crispy,^95^ a graphical interface for multiplet methods implemented in Quanty.^96^ The atomic parameters were scaled to 80% of their Hartree–Fock values. In addition to the atomic term, the crystal field term with a *D*_6h_ symmetry has been included, which is appropriate for the description of U in GO materials. In order to show the effect of the U–O axial bond variation which affects directly the position of the third spectral features and the energy of the 5fσ_u_* orbital, the latter was varied by 0.2 eV increments around the initial value.

Further analysis of HERFD data on TGO, HGO and BGO combined with DFT simulations and EXAFS results suggested that radionuclides predominantly occupy vacancy defects in GO sheets.^93^ The lower sorption of radionuclides by BGO is then explained by the less defected nature of this material compared to other GOs. This conclusion leads to another investigation of the sorption mechanism on defect-rich GO (dGO).^92^ With the help of HERFD in combination with other methods, the mechanism of uranyl sorption by dGO has been defined as essentially the same as in standard HGO, which has a higher number of defects than BGO. At the end, it has been reported that a high abundance of defects in dGO results in a 15-fold increase in the sorption capacity of U(vi) compared to that in standard HGO.^92^

### Speciation, charge compensation and phase separation

One of the first experiments at the U M_4_ edge was performed on a set of mixed uranium oxides – U_4_O_9_ and U_3_O_8_,^3^ where it was shown for the first time that pentavalent U is one of the oxidation states present in these oxides. Few years later, the advances in the principal component analysis (PCA)^75,80,97^ have made it possible to quantify the contribution of the U(v) chemical state in the actinide system. One of the common ways to perform PCA is to use Athena software. However, in order to find the exact composition of different oxidation states in mixed systems, one needs to introduce the spectra of the pure compounds, corresponding to 100% of U(iv), U(v) and U(vi) valence states. This is quite difficult and sometimes impossible, especially in the case of the U(v) valence state in particular coordination. Significant progress has been made by the authors of the iterative transformation factor analysis (ITFA),^97,98^ where the exact composition can be found without the input of the solely separated compounds.

**Fig. 8:**
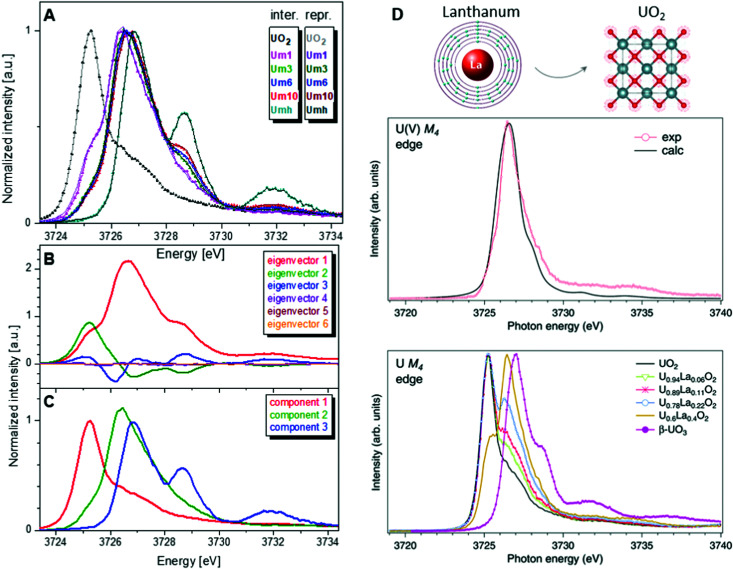
(A) Interpolated experimental (triangles) and reproduced (solid lines) U M_4_ HERFD spectra of the samples with U content of 1000 ppm (Um1), 3000 ppm (Um1), 6000 ppm (Um1) and 10 000 ppm (Um10) formed by co-precipitation of U with magnetite (Fe_3_O_4_) nanoparticles and compared to UO_2_ and 3000 ppm of U(vi) adsorbed onto γ-Fe_2_O_3_ (Umh) serving as reference compounds, from ref. 75. (B) ITFA-extracted eigenvectors; (C) isolated single component spectra belonging to the U(iv), U(v) and U(vi) contributions. (D) HERFD spectra at the U M_4_ edge on UO_2_, U_1−*x*_La_*x*_O_2_ and β-UO_3_ from ref. 74. High-resolution U M_4_ XAS spectrum of U_0.6_La_0.4_O_2_ after subtraction of the U(iv) contribution compared to the calculated XAS spectrum at the M_4_ edge of U(v) in the UO_2_ lattice.^74^

In one of the studies on uranium reduction after U(vi) coprecipitation with magnetite nanoparticles,^75^ the ITFA was used to decompose the spectral mixtures into the spectra and the fractions of the components (Fig. 8). The analysis was carried out in a few steps. First, PCA was performed in order to determine the number of components. It is clearly shown in Fig. 8 that only the first three eigenvectors have a signal, while the following eigenvectors 4–6 do not contribute to the data; hence only three components are necessary to describe the variation in the spectral mixtures. In the beginning, it was considered that the UO_2_ sample contains 100% of the U(iv) valence state, and UO_3_ contains 100% of the U(vi) valence state. The initial analysis showed that the Umh sample contains 100% of the U(vi) valence state. We then used the spectrum of the Umh sample as an input of the 100% of the U(vi) fraction and the UO_2_ sample as 100% of the U(iv) fraction. The iterative target test (ITT) procedure was used in order to find the relative concentrations of the three components. Fig. 8 (left panel, bottom) shows the spectra of the compounds corresponding to U(iv), U(v) and U(vi). Surprisingly the shape of the U(v) contribution has been numerically predicted correctly, due to the good correspondence of the spectral shape of the U(v) compound and the experimental data recorded on NaUO_3_^56^ and KUO_3_.^76^ The analysis showed that all the samples Um1, Um3, Um6 and Um10 contain different concentrations of the U(iv), U(v) and U(vi) valence states, being present simultaneously in each sample.^75^

Such a quantitative empirical analysis can be applied to any spectrum recorded at the U M_4_ edge. In those studies, the ITFA method was applied to the normalized and non-normalized U M_4_ edge data. The fractions derived from both analyses are very similar, with a variation of less than 1%. It should be noted that the crystal structure will influence the energy position of the main HERFD transitions, but as has been discussed already here the difference is in the order of 0.1 eV (*cf.*Fig. 4c) *versus* 1–2 eV between different U oxidation states. Therefore, this confirms the importance of a correct selection of the reference materials during the quantification analysis.

Furthermore, it has been shown that the isolated contribution of the U(v) in the U M_4_ HERFD can be extracted with the help of theoretical simulations. Fig. 8 (right panel) shows the U M_4_ HERFD data^74^ of UO_2_ upon La insertion [U_1−*x*_La_*x*_O_2_(*x* = 0.06, 0.11, 0.22, and 0.4)] which are compared to the UO_2_ and β-UO_3_ references. Inspection of the HERFD data reveals that upon La doping, the shoulder on the high-energy side of the U M_4_ main line of UO_2_ starts to grow and evolves into a dominant peak at 3726.4 eV for U_0.6_La_0.4_O_2_. The energy of this peak, which is in between those of the U M_4_ main lines of the U(iv) and U(vi) references, indicates the U(v) formation. In order to find out the shape of the possible U M_4_ HERFD spectrum of U(v) in an eight-coordination environment, the U M_4_ XANES calculation has been performed using the AIM^83^ approximation. The results are shown in Fig. 8 (right panel), together with U M_4_ HERFD of U_0.6_La_0.4_O_2_ after subtraction of the U(iv) contribution. The experimental and theoretical spectra are in good agreement. By isolating the U(v) contribution to the spectra and by analyzing this contribution in the framework of the AIM, a significant change was found in the characteristics of the chemical bonding in the U(v) subsystem of UO_2_, which must affect the physical and chemical properties of the compound. The charge compensation mechanism and the ability to detect charge changes in actinide compounds are important for fundamental science, as well as for the nuclear industry.

## What we can know

### Defect, non-stoichiometry and doping effects

Ligand K-edge spectroscopy can be considered as a complementary technique to M_4,5_ HERFD, RIXS and XES to probe the electronic structure of the An systems. Together, these methods allow the detailed characterization of An-containing materials at the atomic level. In particular, the ligand K X-ray absorption spectra represent the distribution of the ligand unoccupied p states which are significantly hybridized with An 5f and 6d states and therefore reflect most of the changes in those An states caused by various factors. We show the utility of the method by using an example of the mixed actinide oxide (MOX) study.

MOX is viewed as an innovative fuel for Generation IV reactors which would allow for recycling the major actinides, such as U and Pu, and reducing the radioactive waste by the partitioning and transmutation of the minor ones, such as Np, Am and Cm. The fuel is based on (U,Pu)O_2_ doped with minor actinides. One of the most concerned minor actinides is ^241^Am because of its high radioactivity and significant amount. The goal is to incorporate large quantities of highly radioactive minor actinide into the MOX nuclear fuel. However, only a fluorite type solid solution, as in case of pure (U,Pu)O_2_, needs to be achieved for the final product. For the optimized fuel performance, handling and storage, it is necessary to gain insight into the actinide chemical state and cation charge distribution. In connection with this, the issue of the charge compensation in UO_2_, as a consequence of doping with other actinide and incorporation of the latter into the UO_2_ lattice, becomes also important.

Gaining insight into the cation charge distribution and O/M ratios for mixed oxides is a key parameter to assess the thermodynamic, chemical and physical properties of these fuels. These data are necessary to confirm or improve the existing thermodynamic models and to develop new models and calculations, which are directly linked to the fabrication of these materials. For example, for MOX, the oxygen potential and the O/M ratio are important factors to be considered when designing oxide fuels, because they significantly affect the sintering properties as well as irradiation performances. Therefore, a thorough knowledge of the correlation between the oxygen potential and O/M ratio is essential. To determine this relationship, an accurate experimental determination of the U, Pu and Am oxidation states as a function O/M in the MOX fuel is needed.

(U_1−*y*_Pu_*y*_)O_2_ with a fluorite structure is often slightly non-stoichiometric in oxygen, although the exact O/M ratio (where M = U, Pu) is difficult to establish. In fact, UO_2_ and PuO_2_ show very different oxidation behaviors. At the same time, the local PuO_2_ content in MOX may vary depending on redistribution phenomena linked to the temperature and burn-up profiles in the fuel. The consequent variation in the local oxygen potential can lead to a formation of both anion and cation defects and different types of oxides co-existing with the solid solution matrix, with important consequences on the fuel behavior.

**Fig. 9:**
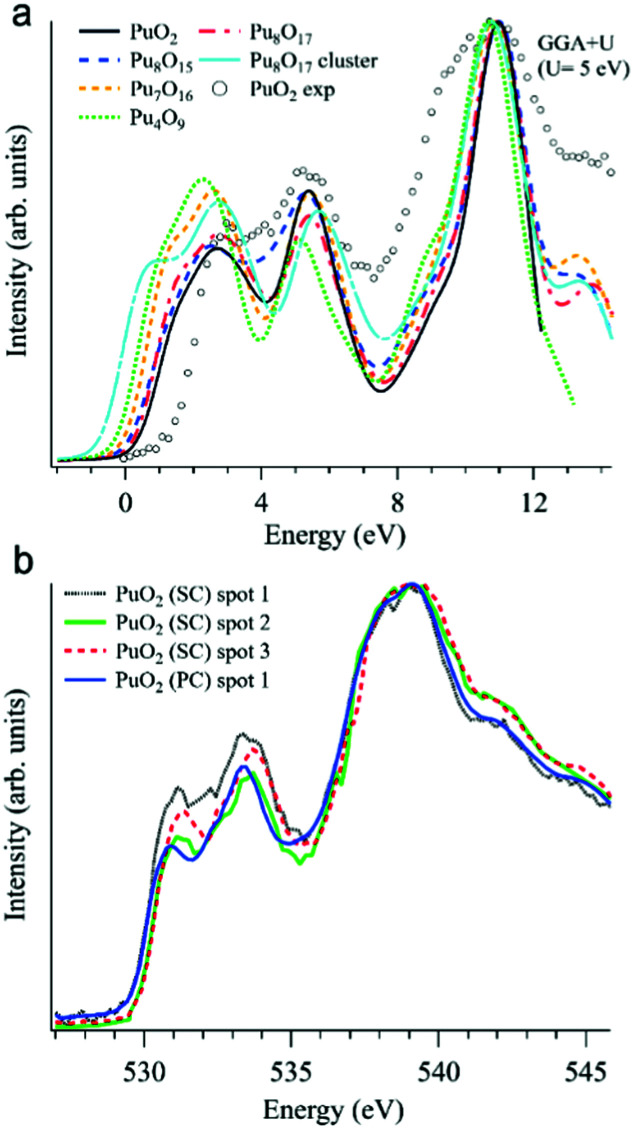
(a) GGA+*U*-calculated O K XAS spectra^99^ of stoichiometric PuO_2_ and various defect structures are shown together with an experimentally recorded spectrum of single crystal PuO_2_ (markers) on the binding energy scale; (b) O K XAS spectra^99^ measured from three spots on the PuO_2_ single crystal (SC) and on polycrystalline (PC) PuO_2_.

In addition to various X-ray spectroscopic methods, measurements at the O K edge is an efficient tool in this type of research.^99–102^ For example, the sensitivity of the O K XAS spectra to the oxygen nonstoichiometry in the PuO_2±*x*_ system was shown^99^ with help of first-principles calculations within the framework generalized gradient approximation with an additional 5f–5f Coulomb interaction *U* taken into account (GGA+*U*). The calculations were performed for the following defect structures: Pu_8_O_15_, Pu_7_O_16_, Pu_4_O_9_, Pu_8_O_17_, and an Pu_8_O_17_ Willis cluster, using *U* = 5 eV for the Coulomb parameter (see Fig. 9a).

Apart from substoichiometric Pu_8_O_15_, which was studied for comparison, the defect structures contain an excess of oxygen. Note that two different kinds of Pu_8_O_17_ structures were calculated to investigate oxygen stoichiometry exceeding that of PuO_2_. When an additional oxygen atom is incorporated in the cubic fluorite structure, the so-called cubo-octahedral interstitial site is known to be energetically the most stable site for the excess oxygen. Willis proposed that fluorite compounds containing an excess anion consist of a defect cluster, the so-called 2 : 1 : 2 Willis cluster, in which the excess oxygen is located at an interstitial site along the (110) direction and the two nearest oxygen atoms are displaced toward the (111) directions, resulting in the creation of two interstitial oxygens and two oxygen vacancies. In this study, Pu_8_O_17_ indicates that the additional oxygen atom is incorporated at a cubo-octahedral interstitial site, whereas the notation Pu_8_O_17_ cluster denotes a defect structure of PuO_2_ containing a 2 : 1 : 2 Willis cluster.

All calculated XAS spectra (Fig. 9a) show a higher intensity of the O 2p–Pu 5f hybridization peak at about 2 eV for the defect structures PuO_2±*x*_ compared to that of stoichiometric PuO_2_, while all but Pu_8_O_15_ reveal a lower intensity in the second peak at ∼5.5 eV which corresponds to the O 2p–Pu 6d hybridization. In particular, the absorption spectra for Pu_8_O_17_ and Pu_4_O_9_ (PuO_2.125_ and PuO_2.25_, respectively) exhibit a growing O 2p–Pu 5f hybridization structure with an increasing oxygen content, yielding the largest increase for Pu_4_O_9_. This results in an increased spectral intensity below 2 eV for the simulated defect structures.

The O K XAS spectra of the aged PuO_2_ single crystal with a Pu-239 isotope were found to be sensitive to self-irradiation effects. The spectra recorded from different areas/spots on the crystal surface (Fig. 9b) show pronounced changes in the shape for the structures at ∼531 eV and 533.4 eV which are attributed to the O 2p–Pu 5f and O 2p–Pu 6d hybridization, respectively, according to the GGA+*U* calculations. The observed changes do not correspond to those predicted by the GGA+*U* calculations for the oxygen excess in the Pu dioxide.

**Fig. 10:**
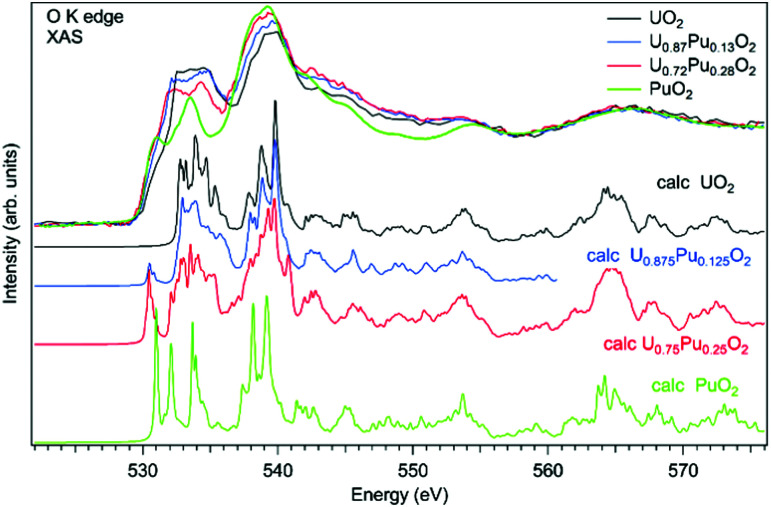
Experimental and calculated O K X-ray absorption spectra of U_1−*x*_Pu_*x*_O_2_.

As an example of actinide doping effects on the electronic structure and the charge distribution/compensation in materials which can be used for the fuel, the results of studies of U_1−*x*_Pu_*x*_O_2_ and U_1−*x*_Am_*x*_O_2_ at the O K XAS edge are shown in Fig. 9 and 10, respectively. The samples were polycrystalline materials prepared using a conventional powder metallurgical technique with the appropriate oxygen potential control. The O K XAS spectra were recorded in the total-fluorescence-yield (TFY) mode at beamline I511 at MAXV-laboratory.

For the U_1−*x*_Pu_*x*_O_2−*y*_ samples, a number of studies of the electronic structure were performed using the X-ray absorption measurements at the U L_3_ and Pu L_3_ edges for both stoichiometric and sub-stoichiometric samples with respect to the oxygen content.^103–106^ The Pu content of the studied materials was in the range from *x* = 0.28 to *x* = 0.50. The studies found uranium and plutonium to be in the U(iv) and Pu(iv) states, respectively, in these samples. Furthermore, the U(iv) and Pu(iv) states were found for both non-irradiated and irradiated U_1−*x*_Pu_*x*_O_2_ materials^107^ with small Pu contents of *x* = 0.04 and 0.03, respectively. On the other hand, X-ray photoelectron spectroscopy (XPS) studies of the U_1−*x*_Pu_*x*_O_2_ thin film^108^ with a high Pu content of *x* = 0.67, which was deposited in low oxygen pressure, detected Pu(iii) on the surface of the sample.

The O K XAS spectra of U_1−*x*_Pu_*x*_O_2_ show significant differences (Fig. 10) when going from pure UO_2_ to pure PuO_2_ as was already described in ref. 99 and 102. This is because the energy splitting between the 5f states which are at the bottom of the conduction band and 6d states increases in PuO_2_. The increasing energy splitting causes the relatively broad 532.5–535 eV structure in UO_2_ to split into two ∼531 eV and 533.4 eV structures in PuO_2_ discussed above. For the U_1−*x*_Pu_*x*_O_2_ system, the observed changes in the O K XAS spectra (Fig. 10) are gradual with the increasing Pu content and that is expected for a situation when tetravalent uranium is substituted by actinide of the same valency, thus confirming that plutonium is in the Pu(iv) state.

The experimental O K XAS spectra are also compared with the results of the calculations using OCEAN which is the *ab initio* density functional theory (DFT) + Bethe–Salpeter equation (BSE) code for the calculations of core-level spectra.^109^ The code allows one to take into account the interaction of the valence-band electrons with the 1s core-hole and screening effects in the calculations of the O K XAS spectra. The DFT+*U* approach was used with the help of Quantum Espresso v. 6.3^110^ and the *U* value was set to 4.5 eV for uranium and to 5.5 eV for plutonium in the U_1−*x*_Pu_*x*_O_2_ system. No spin–orbit interaction for the 5f shell was taken into account since the scalar-relativistic pseudopotentials and local-density-approximation (LDA) functionals were used in calculations. The norm-conserving pseudopotentials were generated using the Opium software package [http://opium.sourceforge.net]. The broadening of the calculated spectra is limited to only the core-hole lifetime to get better understanding of the underlying structures in the experimental spectra. The calculated spectra O K XAS spectra of U_1−*x*_Pu_*x*_O_2_ describe the observed changes in the experimental spectra with Pu doping fairly well and reveal a growing peak at ∼531 eV with the increasing Pu content due to the contribution of the Pu 5f states.

In the case of Am-doped UO_2_, the situation was found to be different. It was reported that americium introduced into the UO_2_ lattice is predominantly in the Am(iii) state.^111–118^ It was argued that the charge compensation process in the U_1−*x*_Pu_*x*_O_2_ system leads to the creation of the U(v) fraction, although for small Am concentrations, it is not easy to confirm that by detecting the U(v) contribution, *e.g.* using the XAS technique at the U L_3_ edge.^112^ Although, the possibility of the existence of complex defects in the oxygen sublattice upon Am doping was discussed^117^ the involvement of oxygen in the charge compensation mechanism in terms of changes in the electronic structure was not analyzed.

**Fig. 11:**
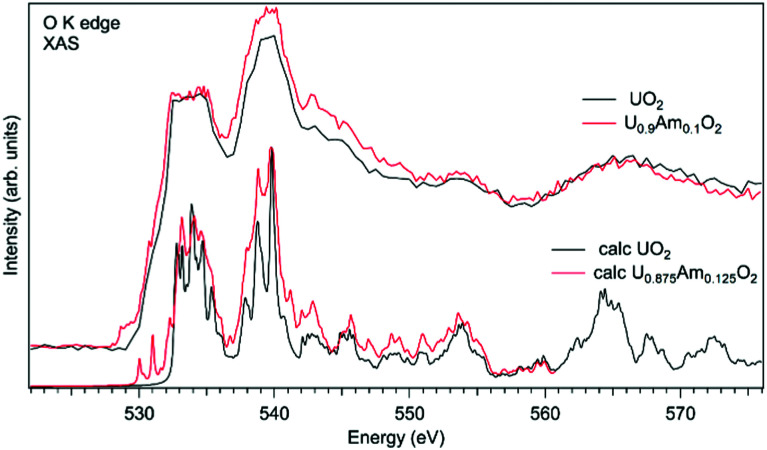
Experimental and calculated O K X-ray absorption spectra of U_1−*x*_Am_*x*_O_2_.

Fig. 11 shows a comparison of the O K XAS spectra of U_0.9_Am_0.1_O_2_ and pure UO_2_. While the main structures in the recorded spectra do not change significantly except for some variations in the relative intensities, the new structure appears at around 529 eV with Am doping. However, the calculations of the O K XAS spectra of U_0.875_Am_0.125_O_2_ (Fig. 11) predict an increased spectral weight at low energies due to the states hybridized with Am 5f contribution but this additional weight is at ∼530 eV and higher. The appearance of a similar ∼529 eV structure was extensively discussed for high-*T*_C_ superconductors, *e.g.* for La_2_CuO_4_ doped with Sr,^119^ and was attributed to the creation of the electronic holes in the O 2p band. It means that for charge compensation of Am(iii) in the UO2 lattice, the electronic holes may be introduced in the O 2p band instead of the creation of U(v). It is also important to note that such electronic holes can be also introduced as a result of the excess of oxygen in the system but in this particular case this is not anticipated due to the control of the oxygen potential at the preparation stage.

Measurements at the ligand K-edge are quite powerful, especially in the sense that new advanced methods and dedicated state-of-the-art instruments have been recently developed at the synchrotron facilities. X-ray Raman scattering (XRS) spectroscopy or non-resonant inelastic X-ray scattering (NIXS) are the new methods to study low energy absorption edges using hard X-rays.^120–125^ The use of hard X-rays makes this technique fundamentally interesting for the actinide science, since K edges of ligands can be probed by hard X-rays without vacuum conditions.^126–130^ Moreover, studies of radioactive samples, sealed in the container, can benefit from the high penetration depth of the hard X-rays.

## Conclusions and perspectives

The electrons in the partially filled outer shell (or the valence shell) determine the chemical properties of the atom. Knowledge about the actinide electronic structure can be obtained by means of X-ray spectroscopy measurements at the U M_4,5_ edges that characterize the U 3d–5f electronic excitation. The transitions at the U M_4,5_ edges lie in the energy range of tender X-rays (*i.e.* 3500–3700 eV) making the experiments challenging to perform due to significant X-ray absorption in air as compared to the measurements at the U L_3_ edge (at ∼17 000 eV). It is also a difficult task to prepare and seal the actinide samples in various containers for the measurements at the U M_4,5_ edges compared to those at the U L_3_ edge. However, the X-ray experiments at the U M_4,5_ edges are more favorable, because the core-hole lifetime broadening of the spectral features is smaller than that at the L_3_ edge, the energy resolution is higher and the 5f states are probed directly through the dipole allowed transitions. A further reduction of the line broadening can be achieved using the HERFD technique, where an X-ray emission spectrometer is employed for data collection.

From the discussion above, it is clear that a significant amount of effort has been made in the past to perform high-energy resolution X-ray spectroscopy on actinide-containing materials, to interpret experimental data and to understand the observed phenomena theoretically. Considerable progress has been achieved from a variety of HERFD/RIXS studies. It has been demonstrated above that HERFD/RIXS spectral features are shaped by the electron–electron interaction and directly depend on the number of available f electrons in the systems, thus allowing the unambiguous detection of the 5f occupancy and the oxidation state of complex actinide systems. In addition, HERFD/RIXS experiments at the actinide M_4,5_ edges allow for probing the crystal field, charge transfer effects, speciation and covalency effects in the crystal and electronic structure of actinides.

Many experimental results are yet to be interpreted, such as the RIXS studies of the intermetallic actinide systems, the XES experiments with the non-monochromatic incident beam (the pink or white beam) at the M_4,5_ edges of actinides and the data related to the nature of the chemical bonding and oxidation state verification in actinide organometallic and coordination complexes.^18–21,68,88,89^ The main problem in the HERFD data analysis is that the width of the spectra, the energy positions of the absorption features and the occupation numbers of the 5f levels are not yet fully understood. There is often considerable disagreement among the valence occupation or the formal charge obtained using different experimental techniques and theoretical models. The term “valence” is related to the number of 5f electrons and the “intermediate valence”, “mixed valence” and “configurational fluctuations” refer to the situation where the actinide ions coexist in two or three 5f configurations in the local structure. Moreover, a general question about how the valence fluctuates is still open. There are cases where actinide ions are placed at different crystallographic positions in the structure and contain either one or another valence state. At the same time, there are systems with identical actinide ions in the crystal structure, but where each atom fluctuates between the two valence states all the time. X-ray spectroscopic methods probe the entire system, and there are difficulties in distinguishing between the two “mixed valence” cases. It is worth mentioning here that the interpretation of the spectroscopic features of the mixed valence systems can be hampered because of the multi-electron configuration interactions, crystal field splitting and other phenomena, which still need to be understood by theory.

On the experimental side, further improvement in the resolution, the detectable count rate and the control of the radiation damage caused by X-rays is highly desirable. To overcome the limitations of the low count rates, improvements in the photon flux of the incoming beam can be made. For example, valence band X-ray emission spectroscopy with a pink/white beam can be realized, but the problem of the X-ray damage of the studied materials will remain a major issue. Interestingly, the spectral resolution does not rely on the monochromacity of the incident X-rays when the X-ray spectrometer is used. At the same time, the improvement of the detection methods of the emitted X-rays will overcome the weak signal limitations. One way is to introduce short working distance X-ray emission spectrometers, by keeping the energy resolution relatively high. The count rate for the emitted energies can be improved by the use of in-vacuum tender X-ray spectrometers^47^ in actinide studies, but it will require a design of additional sample holders, suitable for the radioactive materials.

There is another possibility of increasing the signal-to-noise ratio: by moving the crystal analyzer inside the Rowland cycle. The total energy resolution will decrease, but the sharpening effect of the HERFD data remains strong. In cases where wide emission lines are used for the HERFD data collection, the loss in the instrumental energy resolution has little influence on the resolution of the HERFD spectra due to their core-hole lifetime broadening, while improving the count rate. Obviously future developments of the X-ray emission setups are crucial. The resolution of soft X-ray RIXS experiments is below 100 meV, and the hard/tender X-ray spectroscopy beamlines have to follow the same trend.

In turn, the sensitivity of the O K XAS spectra can be taken advantage of by utilizing X-ray Raman spectroscopy in the hard X-ray energy range or by performing electron energy loss spectroscopy (EELS) using the transmission electron microscopes (TEM) in the home laboratory. Both methods can offer researchers a possibility to study radioactive samples with higher activities and easier sample handling than in cases of measurements with soft X-rays which require an ultra-high vacuum environment.

From a theoretical point of view, much development has resulted from the generalization of different theoretical methods; however, there are many questions that have still been left unanswered. The principal difficulties are the interactions involved in the spectroscopic processes, such as hybridization, electron screening, the electron–electron and electron–phonon interactions.^54^ Most theories sacrifice one or another interaction or parameter in an effort to explain a single experiment, while a comparison between theory and different experimental methods is necessary. There is always a debate regarding which approximation to use in order to account for various phenomena. A single unified theory, combining different theoretical approximations, would result in a major stride forward in the physics and chemistry of f-electron systems.

## Conflicts of interest

There are no conflicts to declare.

## Supplementary Material
